# Utility of 3D facial analysis as a biomarker in rare diseases exploration with hereditary angioedema

**DOI:** 10.3389/fmedt.2026.1857918

**Published:** 2026-09-23

**Authors:** Saumya Jamuar, Richard Palmer, Haur Yueh Lee, Faith Li-Ann Chia, Choo Beng Goh, Stuart Lee, Petra Helmholz, Shermaine Chan, Gareth Baynam

**Affiliations:** 1 Genetics Service, KK Women's and Children's Hospital, Singapore, Singapore; 2 SingHealth Duke-NUS Institute of Precision Medicine, Singapore, Singapore; 3 SingHealth Duke-NUS Genomic Medicine Centre, Singapore, Singapore; 4 School of Earth and Planetary Sciences, Curtin University, Perth, WA, Australia; 5 Department of Dermatology, Singapore General Hospital, Singapore, Singapore; 6 Department of Rheumatology, Allergy and Immunology, Tan Tock Seng Hospital, Singapore, Singapore; 7 Takeda Pharmaceuticals (Asia Pacific) Pte Ltd, Singapore, Singapore; 8 KK Research Centre, KK Women's and Children's Hospital, Singapore, Singapore; 9 Rare Care Centre, Perth Children's Hospital, Perth, WA, Australia; 10 Western Australian Register of Developmental Anomalies, King Edward Memorial Hospital, Perth, WA, Australia

**Keywords:** 3D facial imaging, digital biomarker monitoring, digital phenotyping, hereditary angioedema, rare diseases

## Abstract

**Background:**

People living with rare diseases (PLWRD) often face significant challenges in receiving timely and accurate diagnoses, leading to what is known as a diagnostic odyssey. Digital phenotyping (DP) offers a promising solution by leveraging advanced technology to capture unique digital signatures associated with various rare diseases. This innovative approach not only aids in the identification of these conditions but also facilitates the detection of digital biomarkers (DBM). By utilizing DP and DBMs, we can improve both the diagnosis and management of RDs, ultimately leading to better health outcomes for affected participants.

**Objective:**

To identify whether DBMs can be identified by DP utilizing 3D facial imaging techniques in outpatient settings in participants with RDs.

**Design, setting, and participants:**

This multicentre observational study was conducted in 3 hospitals in Singapore. The eligible participants were male and female RD participants of various age groups. The study duration was 4 years and 8 months.

**Interventions:**

Twenty participants of Chinese genetic ancestry were photographed using a 3D camera. Additionally, two participants with hereditary angioedema (HAE) were photographed during acute stages of disease flare-ups.

**Main outcomes and measures:**

The obtained facial scans of participants (that included participants with HAE in non-acute phase) were plotted using Artificial Intelligence-powered software - Cliniface. The growth curves and facial landmarks obtained were compared against the growth curves of normal RD-unaffected individuals of Chinese genetic ancestry. The two participants with HAE were photographed qualitatively over a longer period of time, and their scans were plotted, yielding growth curves.

**Results:**

Distinct facial markers such as periorbital swelling were identified in two qualitatively assessed HAE participants during flare-up stages. This provides an opportunity to explore and validate whether these facial signatures in a disease condition can be assigned as DBM for HAE.

**Conclusions:**

and relevance: This study explores the utility of 3D facial analysis as a DBM in rare diseases such as HAE. These findings offer preliminary objective evidence that supports existing subjective reports of facial features in the literature. Additionally, while DP's diagnostic capabilities may be limited, it successfully identified DBM, which could facilitate disease monitoring in conditions such as HAE.

## 1 Introduction

People living with rare diseases (PLWRDs) often undergo a prolonged diagnostic odyssey, often lasting more than 4 years from the initial presentation, despite advances in genomic testing capabilities (1). Part of the reason is the sheer number of rare and ultra-rare diseases (RDs), estimated to be >10,000 (2), which makes it impossible for any clinician to be familiar with all of them. In addition, many phenotypic features of these rare diseases overlap with those of other common diseases, often leading to misdiagnosis (3).

Advancements in technologies such as digital phenotyping (DP) using 2D and 3D photogrammetry have been used to identify facial signatures associated with these rare disorders, which in turn helps to reduce this diagnostic odyssey (4). In addition, DP provides an opportunity to identify digital biomarkers (DBM), which can then be used to objectively monitor the progression of a disease and/or response to treatment, both in the clinical trial as well as a real-world setting.

Hereditary angioedema (HAE) is a rare genetic disease characterised by intermittent swelling of the soft tissues due to a defect in the SERPING1 protein. The swelling typically involves the periorbital and perioral areas. However, if the swelling involves the airways, it can be life-threatening and requires immediate medical care. However, patients with HAE often go undiagnosed, and even when diagnosed, due to lack of objective biomarkers to define exacerbation often receive delayed treatment, not only leading to increased anxiety among participants, but also putting them at risk of life threatening complications. In this study, we aimed to understand the role of 3D photography in diagnosis and monitoring of participants with HAE.

RDs are compounded by high mortality rates and significant disability, leading to substantial societal costs (5). Due to the limited number of subjects eligible for clinical studies, typically small, single-arm, non-randomized, and open-label approaches are undertaken (6). Also, the outcome measures are often subjective and can depend on both patient motivation and the rater's assessment (7). Furthermore, outcomes typically represent the patient's condition at a specific point in time, whereas the severity of symptoms can fluctuate over time (8). The challenge of clinical heterogeneity significantly hampers the establishment of reliable clinical endpoints in rare disease trials. Small and fragmented patient populations often present insufficient data, making it challenging to define trustworthy outcome measures. Additionally, the constraints of limited diagnostic tools, challenges in identifying eligible participants, and variability in disease progression collectively lower enrolment rates. This leads to extended study timelines and compromises data integrity, ultimately contributing to a higher failure rate in RD trials compared with trials for more common conditions (9, 10). To improve the success of clinical research in this underserved area, the precision and objectivity of outcome measurements are crucial. Digital BioMarkers (DBM) could address this unmet need to some extent.

Establishing DBM in rare diseases (RD) using non-invasive methods, such as 3D facial imaging, is a novel emerging concept. In this study, we present our exploratory work of an earlier hypothesis concerning the utility of 3D facial analysis as a digital biomarker (4) in those with Chinese, Malay, and Indian genetic ancestry. The primary objective of this study was to determine whether facial measurements in PLWRD, characterized by transient episodes of facial oedema, deviate from ethnically matched established norms. Additionally, we sought to evaluate peri-orbital swelling as a exploratory biomarker for identifying HAE flare-ups.

## 2 Materials and methods

This was a prospective cohort study with two different components. The first component is a quantitative case-control analysis, and the second is a prospective qualitative study.

### 2.1 Participant recruitment

Participants were recruited across three hospitals in Singapore, KK Women's and Children's Hospital (KKH), Singapore General Hospital (SGH), and Tan Tock Seng Hospital (TTSH), under an IRB-approved study (SingHealth CIRB 2021/2419). Participants (or their legal guardians if the participant was under 21 years) consented to participate in the study. The study duration was 4 years and 8 months.

### 2.2 Cohort selection

Participants with clinical and/or genetic diagnosis of HAE were recruited for the study. As HAE is a RD, we expanded our cohort by recruiting participants with other RDs that had similar clinical phenotypic features such as facial swelling and/or facial asymmetry. This included participants with mucopolysaccharidosis, Noonan syndrome, incontinentia pigmenti, velocardiofacial syndrome and achondroplasia.

### 2.3 Digital phenotyping

A 3D facial image of each participant was captured by a member of the study team in Singapore using Vectra M3 (https://www.canfieldsci.com/imaging-systems/vectra-m3-3d-imaging-system/) camera system. These images were analyzed with the Cliniface software program to extract facial landmarks and 58 different measurements (11). The resulting de-identified data were transmitted to the Cliniface team in Western Australia (WA). All participants with HAE were photographed at baseline, and then were photographed serially during exacerbation(s), wherever possible.

The method involved an exploratory analysis of variation across 58 different facial measurements of a disease-affected cohort versus typical (unaffected) reference values derived from regression modelling of the demographically matched (age/sex/genetic ancestry) population. For each measurement variable (some measurements being comprised of several variables corresponding to different spatial dimensions), two-tailed t-testing of the test (RD affected) cohort was performed against the regressed model of the corresponding measurement variable derived from a control (RD unaffected) cohort. A total of 108 different measurement variables were investigated. Data for the control cohort were generated as part of a separate study (12) and were used to regress growth curves for each of the measurement variables against which the test cohort could be compared. The growth curve regression methodology followed that described in Jamuar et al. (12).

Statistical analysis of variation between the test and control cohorts used two-tailed t-testing since it was not possible to state the expected direction of variation in each measurement from its norm with certainty beforehand.

For each measurement variable, the analysis proceeded as follows:

1. The control cohort was regressed to estimate the population mean and standard deviation (SD) across the full age range (0-70 years). Outliers exceeding 3 SD were removed, and regression was then performed again without them to produce a growth curve in accordance with the method described in Jamuar et al. (12).
2. A z-score for each observation (participant) in the test cohort was calculated by comparing to the regressed growth curve at the corresponding age.
3. The test cohort z-scores were assessed for normality (skewness and kurtosis) using Monte Carlo hypothesis testing, and the test was rejected if the z-scores were not normally distributed (*P* < 0.05).
4. The null-hypothesis for the test cohort z-scores was defined as being zero (i.e., equivalent to the regressed mean of the control cohort).
5. A two-tailed t-test was performed with absolute values of the t statistic greater than the critical value of 2.093 (DF =19), denoting rejection of the null hypothesis (a significance level of 0.05). The corresponding *p*-value was found from the cumulative distribution function of the t-distribution.

Testing over such a large number of variables introduces a high likelihood of false positive results due to random chance. Therefore, after deriving *p*-values for all measurements that were not rejected out of hand by normality testing, the complete set was adjusted using the Holm-Bonferroni method to control the family-wise error rate (FWER). The Holm-Bonferroni method was used as it is conservative and does not require the set of variables to be independent from one another (correlations between variables being likely due to different measurements being extracted from very nearby regions of the face across the same set of individuals). All statistical analyses were performed using Python (version 3.12) supported by the SciPy (version 1.16.3) and stats models (version 0.14.5) libraries.

## 3 Results

### 3.1 Demographics

A total of 7 participants with HAE were recruited and photographed during the study period. Out of these, 2 participants had exacerbations and serial photographs were obtained for both participants. There were 20 participants of Chinese, 1 of Malay and 1 Indian genetic ancestries. In addition, we recruited 15 participants of Chinese genetic ancestry with other similar RDs (Table 1).

**Table 1:** Recruited participants of Chinese genetic ancestry with RDs other than hereditary angioedema.

Participant	Age category	Sex	Diagnosis
1.	Paediatric	Male	Mucopolysaccharidosis type II
2.	Paediatric	Male	Achondroplasia
3.	Adult	Female	Incontinentia pigmenti
4.	Adult	Female	Incontinentia pigmenti
5.	Paediatric	Male	Mucopolysaccharidosis Type VI
6.	Paediatric	Male	Mucopolysaccharidosis Type VI
7.	Paediatric	Male	Mucopolysaccharidosis Type VI
8.	Paediatric	Female	Mucopolysaccharidosis Type IV - Morquio A syndrome
9.	Paediatric	Female	Achondroplasia
10.	Paediatric	Female	Velocardiofacial syndrome
11.	Paediatric	Male	Achondroplasia
12.	Paediatric	Male	Noonan syndrome
13.	Paediatric	Male	Noonan Syndrome
14.	Paediatric	Female	Achondroplasia
15.	Paediatric	Male	Noonan Syndrome

Use of 3D facial analysis for diagnostics in HAE and other rare diseases.

The test cohort comprised 20 participants of Chinese genetic ancestry (five participants of Chinese ancestry with HAE and 15 with other RDss as shown in Table 1). The test cohort exhibited wide variation in the degree of facial oedema (or similar features) present. Due to the relatively small test sample size, it was not possible to test for sex differences, and all analyses were performed against measurement growth curves regressed against the combined male and female Chinese genetic ancestry reference dataset.

The objective analysis component of our study with quantitative exploratory comparisons had 108 measurement variables analysed from 58 base measurements. Of the 108, 33 variables were rejected for failing the normality test. *P*-values were calculated for the remaining 75 variables and of these, 11 had *p*-values lower than the significance threshold of 0.05. After FWER correction over the 75 variables, a single one – the Outer Canthal, Nasal Angle retained a significant *p*-value of 0.01 allowing for the null hypothesis of equivalency with the control cohort to be rejected. The 11 most significant results (with Holm-Bonferroni unadjusted *p*-values lower than 0.05) are presented in Table 2. The complete table of results is provided in the supplementary material. Mean effect sizes are given in terms of the standardised (Z) scores with the margin of error (ME) calculated at 95%.

**Table 2:** Statistically significant variables and their values.

Measurement	N (norm)	N (test)	Mean Effect Z	95% ME	*P* (raw)	*P* (adj.)
Outer Canthal, Nasal Angle	876	20	0.87	+/- 0.59	0.000	0.010
Interpupillary Distance	875	20	0.63	+/- 0.53	0.005	0.406
Cutaneous Lower Lip Height	859	20	0.64	+/- 0.77	0.006	0.412
Innercanthal Asymmetry	867	20	−0.56	+/- 0.45	0.013	0.941
Innercanthal Width	877	20	0.54	+/- 0.53	0.017	1.0
Labial Fissure Asymmetry (Y)	876	20	−0.51	+/- 0.43	0.025	1.0
Palpebral Fissure Inclination	878	20	−0.50	+/- 0.28	0.027	1.0
Philtral Asymmetry	867	20	0.50	+/- 0.46	0.029	1.0
Outercanthal Width	878	20	0.49	+/- 0.54	0.031	1.0
Mandibular Width	837	20	0.48	+/- 0.49	0.034	1.0
Innercanthal Asymmetry	864	20	0.48	+/- 0.49	0.034	1.0

The measurements of the Outer Canthal, Nasal Angle from both the control and test cohorts (test observations as black stars) are plotted in Figure 1. Especially in the case of the younger participants in the test cohort, the observed values are generally larger than the age matched regressed mean. While this result is statistically significant, due to the small sample size and variation in the character of the test cohort further investigation using independently collected data is necessary to corroborate this outcome.

**Figure 1:**
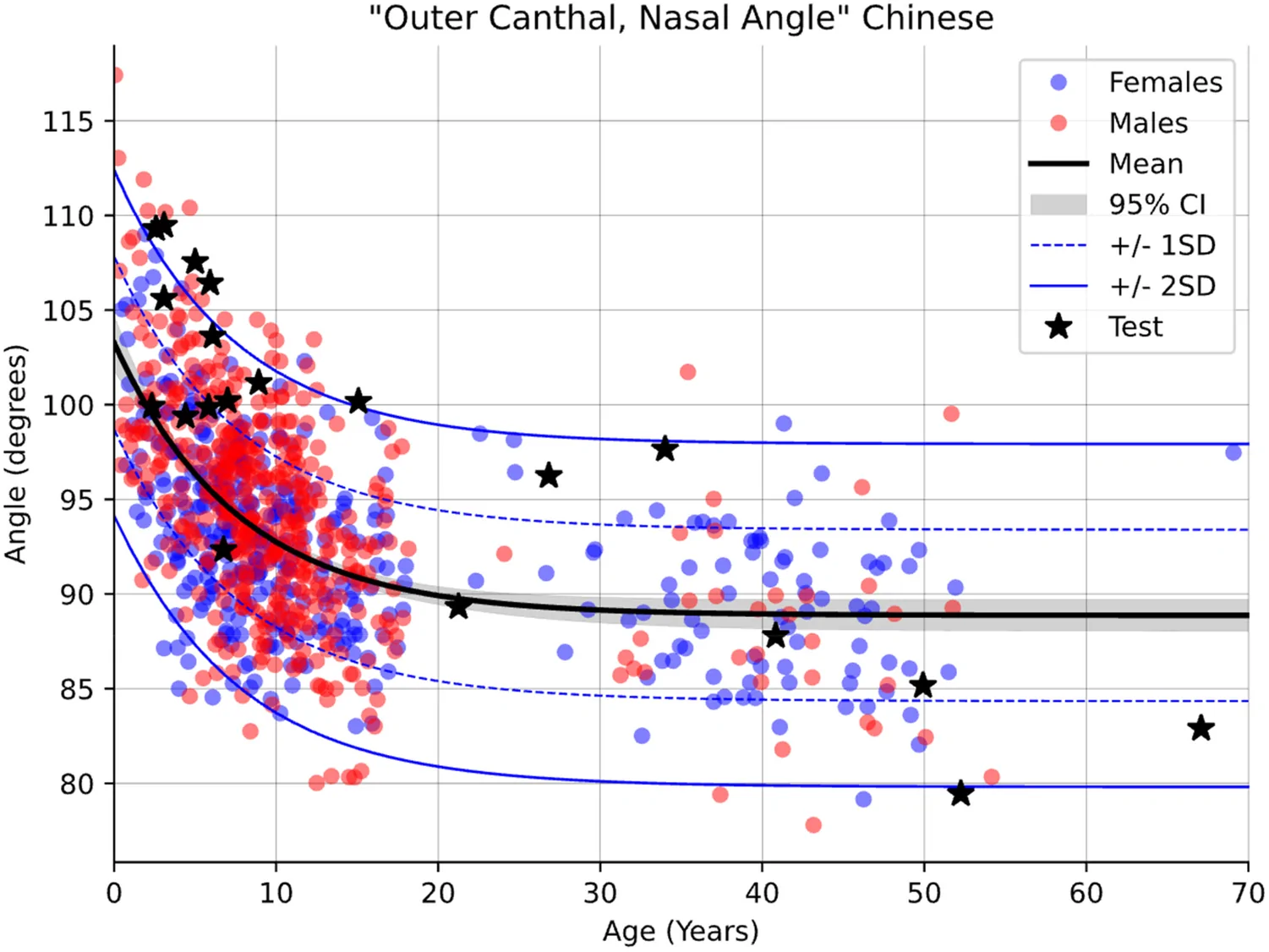
Measurement of the outer canthal, nasal angle in both the control and test cohorts (test observations denoted by black stars).

Of the 75 admissible measurement variables analysed, only the Outer Canthal, Nasal Angle showed a statistically significant difference (after FWER correction) between healthy participants of Chinese genetic ancestry, and those with HAE or other closely mimicking genetic conditions. This suggests that 3D facial imaging may be useful in helping to identify participants who experience facial swelling during remission. Across all other measurements analysed, the observed differences were too limited to yield clear, statistically significant results. Our sample size was small due to the limited population in Singapore and the restricted number of participants with RDs. Apart from the Outer Canthal, Nasal Angle, the results in Table 2 show ten other measurements with small *p*-values - including measurements of specific facial asymmetry - though the significance of these measurements was discounted after bias correction. Further investigation will help to determine if these measurements have utility in being able to objectively indicate the presence of facial oedema (or facial traits presenting similarly). Overall, these results suggest the potential for measurements derived from 3D facial images to monitor resolution of facial oedema in remission and/or detect facial oedema during mild/subclinical exacerbations. Independent follow-up investigations of these measurements will help to provide greater certainty around this study's findings.

### 3.2 Use of 3D facial analysis for monitoring in HAE

A long-term qualitative assessment of serial images of participants with HAE was conducted to identify distinct facial markers associated with the disease. These two different, but complementary, approaches were necessitated by the scarcity of RD cases and the practical complications of capturing facial images of HAE participants during severe and infrequent episodes of acute facial oedema. 3D facial images were captured from two participants over a longer duration to be able to identify markers of facial appearance change likely associated with HAE.

#### 3.2.1 Participant 1

A male, adult participant of Malay genetic ancestry, was photographed at seven time points over 687 days, including images taken immediately after a facial oedema flare (Table 3). Images were imported into Cliniface for Procrustes analysis and visualisation of shape change over time.

**Table 3:** The imaging timelines of the male, adult participant with HAE during qualitative assessment.

Event	Timeline
Baseline 1	Day 1
Baseline 2	Day 154
HAE flare started @ 18:00 – admitted @ 22:00	Day 427
Post 1	Day 428
Post 2	Day 429
Baseline 3	Day 589 (from HAE flare day 1)
Baseline 4	Day 687

*baseline = participant did not complain of exacerbation.

In our analysis of 3D facial images from a male participants with HAE (Figure 2), we initially identified minimal differences in facial volume between the first two baseline measurements taken five months apart (Figure 3). A significant observation was noted during the transition from baseline measurements to the first post-flare assessment: asymmetric peri-orbital swelling, with an increase of more than 3 mm around the right eye. This swelling around the right eye showed some reduction one day later in the second post-flare assessment, while the swelling around the left eye remained unchanged, aside from a slight reduction observed in the left cheek. At the third baseline measurement, taken six months after the flare, the swelling around the right eye had significantly decreased, with a change greater than 3 mm from the second post-flare assessment. There was also a slight increase in swelling around the left eye, which appeared to resolve during the fourth baseline measurement taken three months later (it was later noted that the participant was non-compliant with medication at baseline three, and might have had a mild exacerbation which did not warrant any medical attention). When comparing the second baseline measurement taken 14 months earlier, we found that the periorbital swelling had not completely subsided, although the asymmetry was no longer present. Given the time elapsed since the previous measurements, the findings at baseline four fell within the expected range of variation. Overall, these observations suggest that 3D facial analysis is effective in differentiating between flare and non-flare states, as well as in assessing treatment responses, particularly in instances of non-compliance. Additionally, we noted slight symmetric changes around the cheekbones and lower eyelids, along with a retrusion in the labial fissure, which may have been influenced by the participant's facial expression (slight smile). The final image, captured one day after the second exacerbation, exhibited a similarity in peri-orbital swelling to that seen after the first exacerbation. However, an increase in swelling was observed in the cheeks. The apparent sublabial retrusion is likely an artifact resulting from the average mask fitting being positioned more anteriorly, attributed to the increased extrusion around the eyes and cheeks.

**Figure 2:**
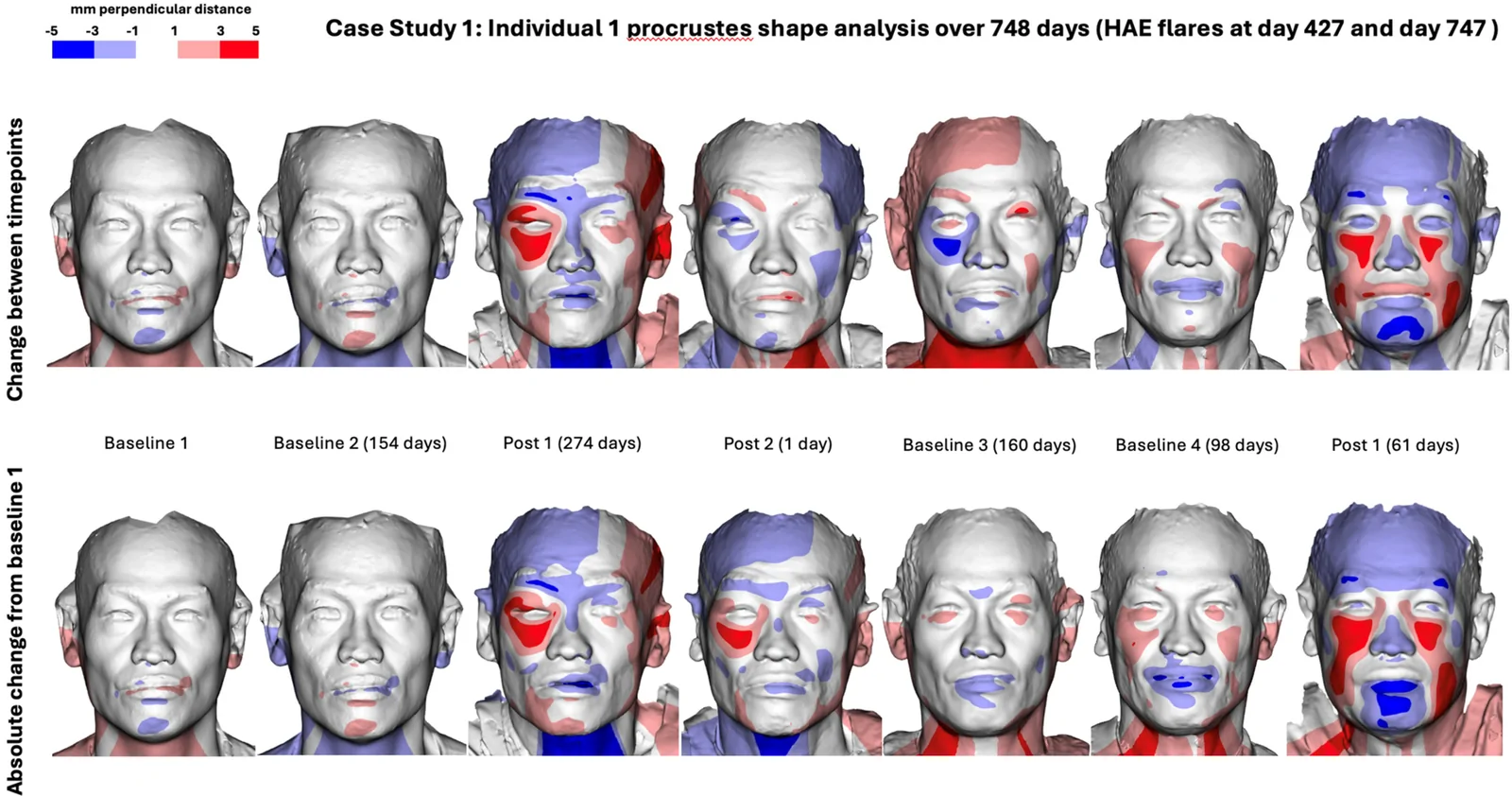
3D facial images of adult male participant with HAE during different stages of diseases over a span of 687 days. The top row compares each image in sequence to its immediately previous one. The bottom row shows a comparison of each image to the initial baseline image (Baseline 1). The colour mapping shows perpendicular extrusion in mm in shades of red, and retrusion in mm in shades of blue.

**Figure 3:**
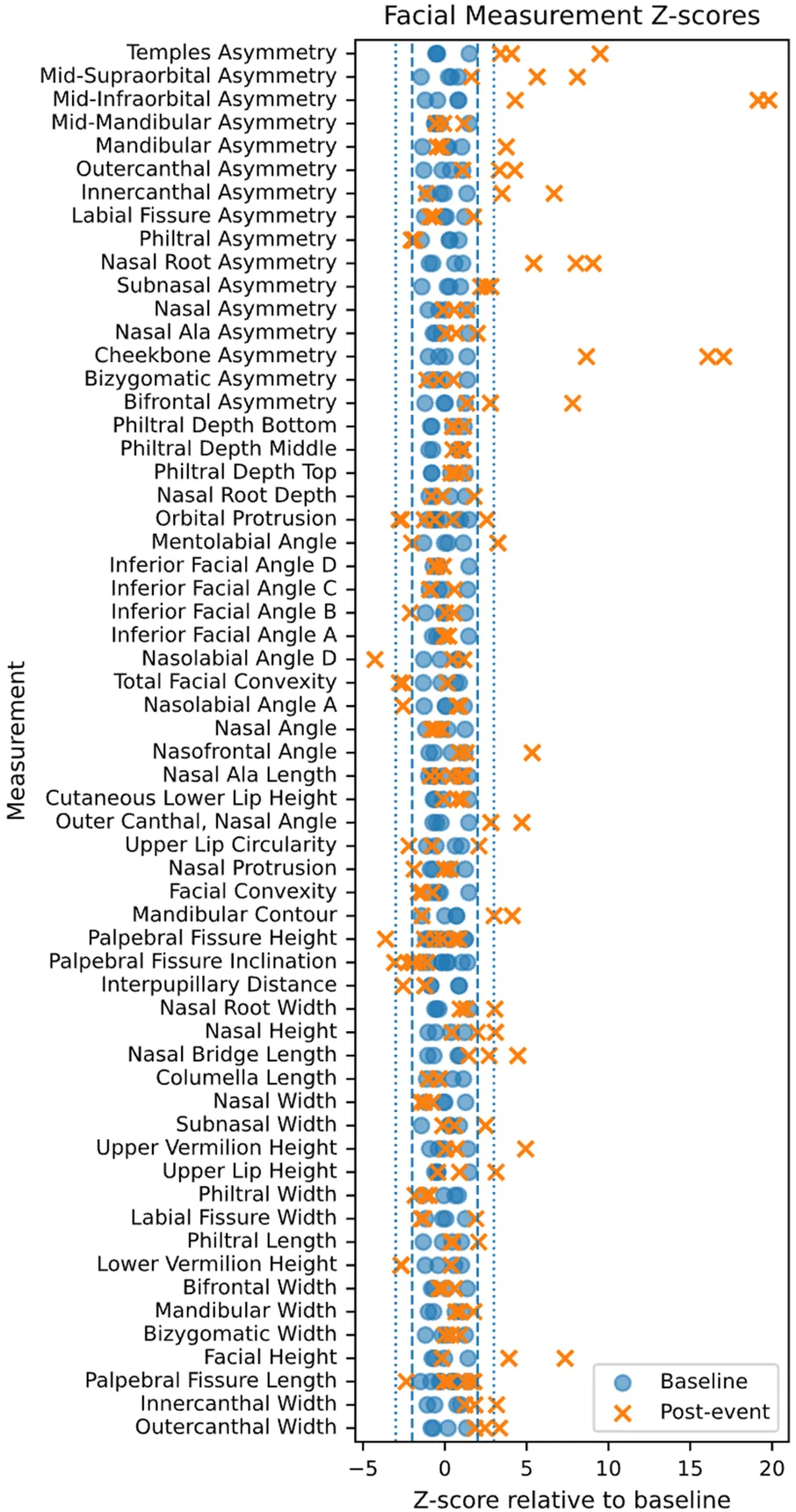
Comparison of facial landmarks and Z-score deviation relative to mean baseline.

Differences between the participant at baseline (4 images) and after experiencing an exacerbation are summarised in Figure 3. Variation in each measurement across the seven datapoints is shown with respect to the mean of the four baseline datapoints.

#### 3.2.2 Participant 2

The second participant was female, adult, of Indian genetic ancestry. She was photographed twice, 261 days apart. The first image was taken immediately after she experienced an acute episode of HAE oedema. A baseline image was taken 261 days later for comparison. The images were imported into Cliniface for procrustes analysis and visualisation of facial shape change between these two timepoints.

The second participant with HAE facial imaging analysis revealed asymmetric peri-orbital swelling (greater on the participant's left). Asymmetric swelling of cheeks and lower jaw – were also more pronounced on participant's left side. Apparent retrusion in midline, and apparent compensatory retrusion in the participant's right upper eyelid seems likely to be an artefact of the average mask fitting (Figure 4). The facial measurement z-scores could not be determined for the second participant as there were only two sets of images for comparison. The asymmetry findings, suggest that approaches that rely only on landmarks, such as crow's feet landmarks, and do not assess symmetry may miss critical indicators of disease flare/ treatment response/ compliance.

**Figure 4:**
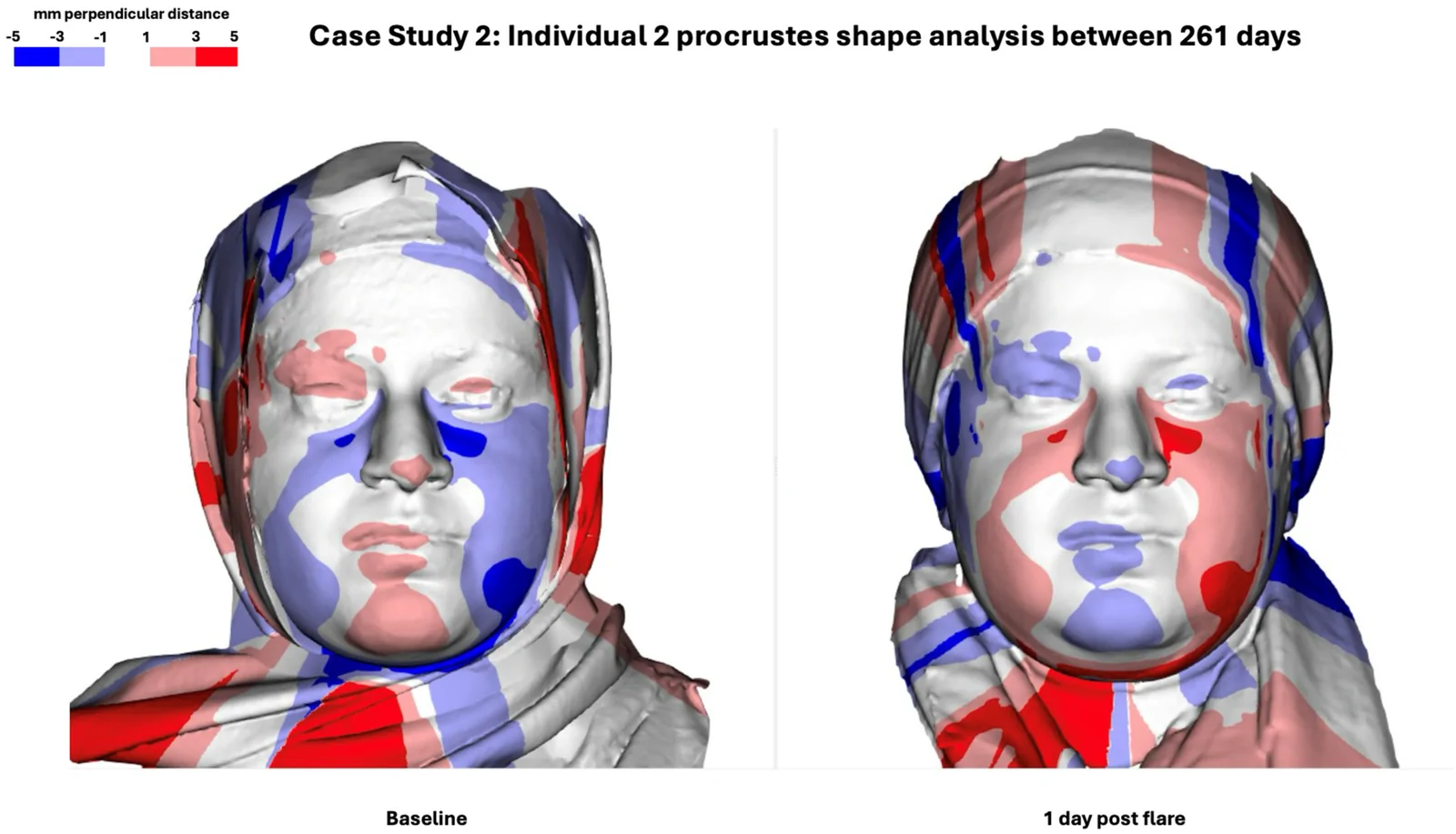
3D facial images of a female participant with HAE at an interval of 261 days. Images are a comparison of one another showing comparative retrusion in mm from the other image/timepoint in shades of blue, and comparative extrusion in mm in shades of red.

## 4 Discussion

Our study conducted an exploratory analysis of facial morphology in participants with RDs, specifically focusing on participants of Chinese genetic ancestry. A total of 20 participants with various genetic conditions associated with facial oedema were compared to a demographically matched control cohort using 3D facial photogrammetry. Facial images were analysed for 58 base measurements, generating 108 measurement variables, demonstrating opportunities to explore, identify, and differentiate syndrome-specific apparent and subtle changes in microdetails. These changes need to be established for their distinct and identifiable signatures during evolutionary or resolutory phases of different RDs and in common diseases. The Supplementary Table 1 enumerates all the tested variables which shows that our study was rigorous and we took into consideration only robust indicators from participant groups. Wider-scale studies need to be conducted on healthy individuals and RD participants hailing from different genetic ancestries, genders, and age groups for comparisons and harmonizations of clinically significant changes associated as identifiable signatures for specific syndromes.

Our study's results demonstrate findings in symmetric and asymmetric facial features that correlate with disease flare, resolution and treatment non-compliance, and that overlap between the imaged participants. This suggests that 3D facial analysis using the Cliniface software may be useful in discriminating HAE flare (acute episode) from non-flare states. The nature of the swelling shown in the post-flare images in both individuals is similar. Both participants have notable asymmetric peri-orbital swelling (though on different sides), and swelling of the cheeks and lower jaw is also apparent in both. From these observations we can provisionally interpret that periorbital swelling and asymmetry during acute episode of HAE through 3D facial imaging could be a potential DBM to predict the course of the disease as working hypothesis.

Even in preliminary finding the results of this study are limited by the small sample size and inherent variability in the test cohort due to incorporation of different disease types. Larger studies with diverse participants is important to demonstrate applicability of these results across different populations. However, given that we used the participants as their control, DBM identified through this study are tentative inference and can be explored further as DP in HAE.

DP and DBM identified through 3D facial analysis, for disease screening, early disease detection, monitoring, and personalized treatment are a step towards non-invasive measurement approaches (13). Our study provides an exploratory utility of 3D facial imaging as a DBM in RDs. While challenges such as small sample sizes and limited genetic ancestry diversity persist, ongoing data collection and technological advancements require further broad-based investigations, validation, and applications. Cliniface's precision underscores the potential of digital tools in advancing precision medicine and improve patient outcomes.

## 5 Limitations

Our study has several shortcomings. The major limitation of the study is a very small and highly heterogeneous cohort. Only 20 participants in our comparative study were of Chinese genetic ancestry, and of these only five of these individuals had HAE. The relatively small test sample size also meant that normality testing of the variables was low in statistical power increasing uncertainty in the analytical results. A large and diverse pool of participants and comparison across different ethnic groups is crucial for increasing confidence in the results obtained. In a Chinese genetic ancestry majority country such as Singapore, with a small population base, this was inevitable. Only five Chinese participants with HAE were included in the quantitative analysis, while the remaining participants had unrelated syndromic disorders such as achondroplasia, Noonan syndrome, mucopolysaccharidosis, and velocardiofacial syndrome. The heterogeneity made our comparisons less than ideal but given the overlap in some of the features we attempted a comparison. But for the two HAE patients, we have kept the analysis confined to the two HAE patients where we do have follow up photographs, using them as their own control. The normal subject comparison arm comprised mainly paediatric subjects, while the diseased state participant group included some adults also. Our qualitative analysis of participants with HAE was limited to two participants only. Whilst the HAE participant numbers were small, the use of multiple images per affected participant and the consistency of findings between the affected participants (swelling, asymmetry) are supportive and biologically plausible. An additional critical limitation involves demographic matching. While the manuscript highlights ethnicity-specific growth curves, it does not account for sex differences, and the diseased cohort comprises both paediatric and adult participants. These demographic variables may significantly affect craniofacial measurements and are potential sources of bias. We hope to further establish our experimental findings through ongoing data collection from participants of different genetic ancestries and disease conditions to identify additional DBMs.

## 6 Conclusion

Our study provides an exploratory utility of 3D facial imaging as a DBM in RDs. We have exhibited preliminary and tentative approach (quantitative and qualitative) for establishing efficacy of DBM from 3D facial images for objective detection of latent facial oedema To confidently assert the clinical utility of our approach for monitoring, a larger number of cases need to be evaluated, rigorous repeatability testing must be conducted, and well-defined performance metrics distinguishing between flare and non-flare states are essential. These important steps would be instrumental in providing robust evidence for the method's reliability and effectiveness in real-world clinical settings.

## Data Availability

No. Participant-level data on which the analysis was based will not be shared to protect participant anonymity.

## Data availability statement

The datasets presented in this article are not readily available because participant-level data on which the analysis was based will not be shared to protect participant anonymity. Requests to access the datasets should be directed to Gareth.Baynam@health.wa.gov.au.
