# Supplementary material for: Utility of 3D facial analysis as a biomarker in rare diseases exploration with hereditary angioedema
Source: Front Med Technol. 2026 Sep 23;8:1857918. doi: 10.3389/fmedt.2026.1857918 (PMC13642716; doi:10.3389/fmedt.2026.1857918)
Supplement: Supplementary file 1 [file Table1.docx]

**Supplementary table.** Results for all tested variables (rejected samples not shown).

| **Measurement** | **Dimension** | **N (norm)** | **N (test)** | **Effect Z** | **+/- 95% ME** | **P (raw)** | **P (adj.)** |
| --- | --- | --- | --- | --- | --- | --- | --- |
| Outercanthal Width | 0 | 878 | 20 | 0.4907 | 0.5373 | 0.0308 | 1 |
| Innercanthal Width | 0 | 877 | 20 | 0.5407 | 0.5279 | 0.0173 | 1 |
| Palpebral Fissure Length | 0 | 878 | 20 | 0.1712 | 0.5708 | 0.4517 | 1 |
| Facial Height | 0 | 859 | 20 | 0.2282 | 0.4976 | 0.3138 | 1 |
| Bizygomatic Width | 0 | 852 | 19 | -0.0807 | 0.5676 | 0.7292 | 1 |
| Mandibular Width | 0 | 837 | 20 | 0.4816 | 0.488 | 0.0336 | 1 |
| Philtral Length | 0 | 875 | 20 | 0.369 | 0.5286 | 0.104 | 1 |
| Labial Fissure Width | 0 | 878 | 20 | -0.2708 | 0.4206 | 0.2299 | 1 |
| Philtral Width | 0 | 875 | 20 | -0.2767 | 0.4022 | 0.2195 | 1 |
| Nasal Width | 0 | 878 | 20 | 0.1002 | 0.4756 | 0.6576 | 1 |
| Columella Length | 0 | 876 | 20 | -0.2105 | 0.5208 | 0.3531 | 1 |
| Nasal Bridge Length | 0 | 877 | 20 | -0.3908 | 0.5235 | 0.085 | 1 |
| Nasal Height | 0 | 877 | 20 | -0.1139 | 0.5042 | 0.6148 | 1 |
| Nasal Root Width | 0 | 875 | 20 | 0.2865 | 0.4928 | 0.206 | 1 |
| Interpupillary Distance | 0 | 875 | 20 | 0.6312 | 0.5252 | 0.0055 | 0.4064 |
| Palpebral Fissure Inclination | 0 | 878 | 20 | -0.4967 | 0.2774 | 0.0269 | 1 |
| Palpebral Fissure Height | 0 | 875 | 20 | -0.2751 | 0.5595 | 0.2263 | 1 |
| Mandibular Contour | 0 | 833 | 20 | -0.3171 | 0.5153 | 0.1622 | 1 |
| Facial Convexity | 0 | 848 | 20 | -0.0292 | 0.5053 | 0.8976 | 1 |
| Nasal Protrusion | 0 | 875 | 20 | 0.145 | 0.5583 | 0.5235 | 1 |
| Upper Lip Circularity | 0 | 864 | 20 | -0.0355 | 0.48 | 0.8749 | 1 |
| Outer Canthal, Nasal Angle | 0 | 876 | 20 | 0.8722 | 0.5869 | 0.0001 | 0.0102 |
| Cutaneous Lower Lip Height | 0 | 859 | 20 | 0.6408 | 0.7703 | 0.0056 | 0.4124 |
| Nasal Ala Length | 0 | 874 | 20 | -0.0625 | 0.5027 | 0.7826 | 1 |
| Nasofrontal Angle | 0 | 864 | 20 | -0.1492 | 0.3825 | 0.5081 | 1 |
| Nasal Angle | 0 | 875 | 20 | 0.3645 | 0.4489 | 0.1067 | 1 |
| Nasolabial Angle A | 0 | 877 | 20 | 0.1583 | 0.5103 | 0.4854 | 1 |
| Total Facial Convexity | 0 | 847 | 20 | 0.3183 | 0.5486 | 0.1619 | 1 |
| Inferior Facial Angle A | 0 | 842 | 20 | -0.2856 | 0.4984 | 0.2082 | 1 |
| Inferior Facial Angle B | 0 | 856 | 20 | -0.0599 | 0.5459 | 0.7922 | 1 |
| Inferior Facial Angle C | 0 | 837 | 20 | 0.21 | 0.5585 | 0.3564 | 1 |
| Inferior Facial Angle D | 0 | 850 | 20 | 0.4069 | 0.6593 | 0.0762 | 1 |
| Mentolabial Angle | 0 | 856 | 20 | -0.1464 | 0.5968 | 0.5204 | 1 |
| Philtral Depth | 0 | 877 | 20 | -0.124 | 0.5009 | 0.5843 | 1 |
| Philtral Depth | 1 | 876 | 20 | -0.0319 | 0.5538 | 0.8888 | 1 |
| Philtral Depth | 2 | 872 | 20 | -0.2079 | 0.414 | 0.3566 | 1 |
| Orbital Protrusion | 0 | 802 | 20 | -0.0893 | 0.543 | 0.6946 | 1 |
| Nasal Root Depth | 0 | 878 | 20 | -0.3203 | 0.3845 | 0.1549 | 1 |
| Bifrontal Asymmetry | 0 | 802 | 20 | 0.0524 | 0.3815 | 0.816 | 1 |
| Bizygomatic Asymmetry | 0 | 852 | 19 | -0.185 | 0.3501 | 0.4227 | 1 |
| Bizygomatic Asymmetry | 2 | 846 | 19 | -0.1392 | 0.3932 | 0.5471 | 1 |
| Cheekbone Asymmetry | 0 | 870 | 20 | -0.3724 | 0.7641 | 0.1073 | 1 |
| Cheekbone Asymmetry | 1 | 867 | 20 | -0.0471 | 0.7381 | 0.8382 | 1 |
| Nasal Ala Asymmetry | 0 | 870 | 20 | -0.3983 | 0.3739 | 0.0773 | 1 |
| Nasal Asymmetry | 0 | 870 | 20 | -0.3825 | 0.3873 | 0.0899 | 1 |
| Nasal Asymmetry | 1 | 874 | 20 | -0.2155 | 0.4386 | 0.34 | 1 |
| Subnasal Asymmetry | 0 | 873 | 20 | -0.3666 | 0.3218 | 0.103 | 1 |
| Subnasal Asymmetry | 1 | 877 | 20 | -0.1156 | 0.3406 | 0.6071 | 1 |
| Subnasal Asymmetry | 2 | 877 | 20 | 0.1301 | 0.5058 | 0.5663 | 1 |
| Nasal Root Asymmetry | 0 | 868 | 20 | -0.1531 | 0.3308 | 0.4963 | 1 |
| Nasal Root Asymmetry | 1 | 874 | 20 | 0.0227 | 0.5349 | 0.9201 | 1 |
| Nasal Root Asymmetry | 2 | 873 | 20 | 0.3059 | 0.3192 | 0.1737 | 1 |
| Nasal Root Asymmetry | 3 | 867 | 20 | -0.0778 | 0.2906 | 0.7293 | 1 |
| Philtral Asymmetry | 0 | 871 | 20 | -0.1806 | 0.5406 | 0.4268 | 1 |
| Philtral Asymmetry | 1 | 873 | 20 | -0.2459 | 0.5167 | 0.2787 | 1 |
| Philtral Asymmetry | 2 | 873 | 20 | -0.2498 | 0.4693 | 0.2698 | 1 |
| Philtral Asymmetry | 3 | 867 | 20 | 0.4946 | 0.4634 | 0.029 | 1 |
| Labial Fissure Asymmetry | 0 | 870 | 20 | -0.3674 | 0.4895 | 0.1051 | 1 |
| Labial Fissure Asymmetry | 1 | 876 | 20 | -0.5067 | 0.4335 | 0.025 | 1 |
| Labial Fissure Asymmetry | 3 | 869 | 20 | 0.2198 | 0.4612 | 0.3312 | 1 |
| Innercanthal Asymmetry | 0 | 867 | 20 | -0.5621 | 0.4489 | 0.0131 | 0.9406 |
| Innercanthal Asymmetry | 1 | 875 | 20 | -0.3141 | 0.4972 | 0.166 | 1 |
| Innercanthal Asymmetry | 2 | 875 | 20 | -0.0833 | 0.5537 | 0.7142 | 1 |
| Innercanthal Asymmetry | 3 | 864 | 20 | 0.4804 | 0.4865 | 0.0342 | 1 |
| Outercanthal Asymmetry | 0 | 870 | 20 | 0.0883 | 0.4454 | 0.6961 | 1 |
| Outercanthal Asymmetry | 1 | 873 | 20 | 0.0935 | 0.5848 | 0.6815 | 1 |
| Outercanthal Asymmetry | 2 | 870 | 20 | 0.1949 | 0.396 | 0.3876 | 1 |
| Outercanthal Asymmetry | 3 | 867 | 20 | 0.2425 | 0.3527 | 0.2813 | 1 |
| Mid-Infraorbital Asymmetry | 0 | 866 | 20 | 0.1669 | 0.7722 | 0.4704 | 1 |
| Mid-Infraorbital Asymmetry | 1 | 866 | 20 | -0.2778 | 0.5439 | 0.2218 | 1 |
| Mid-Infraorbital Asymmetry | 2 | 865 | 20 | 0.3724 | 0.3786 | 0.0986 | 1 |
| Mid-Infraorbital Asymmetry | 3 | 861 | 20 | 0.3412 | 0.4677 | 0.1317 | 1 |
| Mid-Supraorbital Asymmetry | 0 | 871 | 20 | 0.1483 | 0.5951 | 0.5156 | 1 |
| Mid-Supraorbital Asymmetry | 2 | 868 | 20 | 0.2434 | 0.4328 | 0.2813 | 1 |
| Temples Asymmetry | 1 | 797 | 20 | 0.1316 | 0.3181 | 0.5583 | 1 |
